# A small-molecule TLR4 antagonist reduced neuroinflammation in female E4FAD mice

**DOI:** 10.1186/s13195-023-01330-6

**Published:** 2023-10-19

**Authors:** Deebika Balu, Ana C. Valencia-Olvera, Austin Nguyen, Mehul Patnam, Jason York, Francesco Peri, Frank Neumann, Mary Jo LaDu, Leon M. Tai

**Affiliations:** 1 Department of Anatomy and Cell Biology, University of Illinois at Chicago, Chicago, IL 60612 USA; 2 Department of Biotechnology and Biosciences, University of Milano-Bicocca, Milan, Italy; 3 Innaxon Biosciences, Tewkesbury, UK

**Keywords:** Alzheimer’s disease, Neuroinflammation, TLR4, EFAD, IAXO-101, Treatment paradigms, Apolipoprotein E, Female sex

## Abstract

**Background:**

*APOE* genotype is the greatest genetic risk factor for sporadic Alzheimer’s disease (AD). *APOE4* increases AD risk up to 12-fold compared to *APOE3*, an effect that is greater in females. Evidence suggests that one-way *APOE* could modulate AD risk and progression through neuroinflammation. Indeed, *APOE4* is associated with higher glial activation and cytokine levels in AD patients and mice. Therefore, identifying pathways that contribute to *APOE4*-associated neuroinflammation is an important approach for understanding and treating AD. Human and in vivo evidence suggests that TLR4, one of the key receptors involved in the innate immune system, could be involved in *APOE*-modulated neuroinflammation. Consistent with that idea, we previously demonstrated that the TLR4 antagonist IAXO-101 can reduce LPS- and Aβ-induced cytokine secretion in *APOE4* glial cultures. Therefore, the goal of this study was to advance these findings and determine whether IAXO-101 can modulate neuroinflammation, Aβ pathology, and behavior in mice that express *APOE4*.

**Methods:**

We used mice that express five familial AD mutations and human *APOE3* (E3FAD) or *APOE4* (E4FAD). Female and male E4FAD mice and female E3FAD mice were treated with vehicle or IAXO-101 in two treatment paradigms: prevention from 4 to 6 months of age or reversal from 6 to 7 months of age. Learning and memory were assessed by modified Morris water maze. Aβ deposition, fibrillar amyloid deposition, astrogliosis, and microgliosis were assessed by immunohistochemistry. Soluble levels of Aβ and apoE, insoluble levels of apoE and Aβ, and IL-1β were measured by ELISA.

**Results:**

IAXO-101 treatment resulted in lower Iba-1 coverage, lower number of reactive microglia, and improved memory in female E4FAD mice in both prevention and reversal paradigms. IAXO-101-treated male E4FAD mice also had lower Iba-1 coverage and reactivity in the RVS paradigm, but there was no effect on behavior. There was also no effect of IAXO-101 treatment on neuroinflammation and behavior in female E3FAD mice.

**Conclusion:**

Our data supports that TLR4 is a potential mechanistic therapeutic target for modulating neuroinflammation and cognition in *APOE4* females.

**Supplementary Information:**

The online version contains supplementary material available at 10.1186/s13195-023-01330-6.

## Background

*APOE* (apolipoprotein E) genotype is a major risk factor for sporadic Alzheimer’s disease (AD), with *APOE4* increasing risk up to 12-fold compared to *APOE3* (reviewed in [1, 2]). Therefore, an important challenge is identifying therapeutic targets for *APOE4* carriers in AD. The role of *APOE* in AD is complex, as *APOE* has been shown to modulate multiple functions and pathways in humans and transgenic mouse models that overproduce amyloid-beta (Aβ) via familial AD mutations (FAD) (reviewed in [3, 4]). For example, compared to *APOE3*, *APOE4* is associated with higher soluble Aβ levels and amyloid plaques, altered metabolism, and neurovascular function (reviewed in [3–5]). In addition, increasing evidence suggests that *APOE*-modulated neuroinflammation contributes to AD progression [6]. In AD patients, compared to *APOE3*, *APOE4* is associated with earlier onset of cognitive deficits and increased neuroinflammation [7–10]. These human data are recapitulated in vivo, as *APOE4* is associated with greater microgliosis [11–14], astrogliosis [13–15], and altered cytokine levels [12, 14, 16, 17] in FAD mice and in *APOE*-knock in mice after induction of peripheral inflammation. In addition, there is greater neuroinflammation in female *APOE4* FAD mice compared to males [11, 15, 18, 19], consistent with higher AD risk and pathology in female *APOE4* carriers. Therefore, identifying pathways that contribute to *APOE4*-associated neuroinflammation is an important approach for developing AD therapeutics.

Neuroinflammation is complex and involves multiple cell-types, receptors, signaling pathways, cytokines, and chemokines. One approach to identify the contribution of neuroinflammatory pathways to AD progression is to evaluate activity of FDA approved drugs with known anti-inflammatory activities such as NSAIDs [20]. Although some studies supported that NSAIDs may be efficacious as an AD therapeutic, including in *APOE4* carriers, others have shown no beneficial effects [21–23]. Ongoing research is defining if these established anti-inflammatories will be beneficial for specific patient groups at certain stages of AD and the optimal treatment regime. An alternative approach is to determine if compounds that target pathways modulated by *APOE4* can impact neuroinflammation and behavior. The innate immune system is one of the most highly conserved immune responses across plants, drosophila, and animals to defend against invading pathogens and is also activated by endogenous stress-related molecules. Toll-like receptor 4 (TLR4) is a key component of the innate immune response is expressed by microglia (and to a lesser extent by astrocytes) [24, 25] and is activated by lipopolysaccharide (LPS) and other stress-associated ligands (reviewed in [26, 27]). Evidence suggests that TLR4 could contribute to neuroinflammation in AD. For example, there is higher TLR4 expression in AD patients’ [28, 29] and FAD mouse brains [29, 30] and single nucleotide polymorphisms (SNPs) in TLR4 have been shown to modulate AD risk [31–33]. There is also evidence that TLR4 is involved in *APOE4*-associated neuroinflammation. In vitro, LPS-induced inflammatory response is greater with *APOE4* compared to *APOE3* [14, 16, 17]. Furthermore, Aβ-induced cytokine production is greater with *APOE4* in glial cultures, an effect that is blocked by TLR4 antagonists, and expression of TLR4-related genes are greater in *APOE4*-FAD mice [34]. Based on these data, it has been proposed that with *APOE4*, greater TLR4 activation in the brain may lead to higher neuroinflammatory responses and contribute to behavioral deficits. However, to date, no studies have directly evaluated the effect of blocking TLR4 on AD-relevant pathology and behavior in mice that express *APOE4*.

The goal of this study was to determine whether TLR4 antagonism can modulate neuroinflammation, Aβ pathology, and behavior in mice that express *APOE4*. To address this goal, we used a novel small molecule TLR4 antagonist IAX0-101, which we have previously shown to inhibit Aβ-induced cytokine release in *APOE4*-mixed glial cells [34]. Therefore, we treated mice that express *APOE4* (E4FAD) and overproduce Aβ with IAXO-101 in prevention and reversal paradigms and evaluated the impact on neuroinflammation, Aβ pathology, and behavior.

## Methods

### Animals and study design

All experiments follow the University of Illinois at Chicago Animal Care Committee protocols. EFAD mice express five familial AD mutations and human *APOE* (5xFAD^+/-^/h*APOE*^+/+^) as described in [35]. EFAD mice were generated from Tg6799, the 5xFAD mouse strain that produced the highest amount of Aβ42. In Tg6799 5xFAD mice, Aβ40 levels also increase with age but rise more slowly and are substantially lower than for Aβ42 in young mice [36]. The prevention (PVT) treatment paradigm was designed to begin at 4 months, at early stages of AD pathology, including amyloid deposition and neuroinflammation, and end at 6 months when pathology is significant [35]. The reversal (RVS) treatment paradigm begins at 6 months and ends at 7 months, capturing the previously observed age-associated increase in pathology [37].

### Drug formulation and treatment

IAXO-101, a synthetic Cluster of differentiation 14 (CD14)/TLR4 antagonist nano-formulated in Lipodisq™, was provided by Innaxon Biosciences (Tewkesbury, UK). Lipodisq™ drug formulations are lipid-based nano-sized (10-40 nm) monodisperse, discoidal nanoparticles, also referred to as native nano-discs or styrene maleic acid lipid particles [38]. EFAD mice were administered either IAXO-101 in Lipodisq™ nano-formulation or a vehicle (empty Lipodisq™ nano-formulation) using subcutaneous injections at 10 mg/kg for three times per week. The nano-formulated IAXO-101 was provided as a sterile, endotoxin-tested, 4 mg/ml aqueous stock solution, diluted just prior to use in sterile, endotoxin-free water [38, 39]. Treatments for the mice were randomized within cage and across groups. All investigators were blinded for treatment and analysis. Body weights were measured prior to each injection to determine dose and monitor for any treatment-related weight changes.

### Behavioral analysis and tissue harvest

All behavioral data were recorded and analyzed with ANY-maze video tracking software (Stoelting Co, Wood Dale, IL USA). In the week prior to sacrifice, mouse behavior was tested using a modified Morris water maze protocol with acquisition trials consisting of 4 × 1 min trials/day for 5 consecutive days with latency to the platform recorded for each trial. A single probe trial was run on day 6 with the platform removed, and the readouts included latency to platform and latency to target quadrant (previously described [40–42]). After the probe trial, the mice were anesthetized with ketamine/xylazine and perfused with phosphate-buffered saline. Then, the brains were removed and dissected at the midline to produce two hemi-brains, one each for immunohistochemical and biochemical analysis (previously described in [35, 42]).

### Immunohistochemical analysis

Serial sagittal brain sections (35 μm thick, 280 μm apart) from EFAD mice were all stained for fibrillar amyloid deposition via Thio-S and immunostained for Aβ deposition, astrogliosis, and microgliosis (previously described [11, 14, 35]), with anti-mouse or anti-rabbit Alexa-fluor secondary antibodies. A list of all the antibodies used in this study is provided in Additional file 2. The stained sections were imaged at × 10 magnification with a Zeiss Fluorescence microscope and analyzed for area covered by Thio-S, MOAB-2, GFAP, S100β, C3, Iba-1, and Clec7a in the cortex (CX) and hippocampus (HP) using ImageJ. The regions included for analysis are outlined in Fig. S1A (CX = 1; HP = 2 + 3) and close-up images of CX and HP at × 20 for all immunostainings performed are presented in Additional file 1 (Figs. S8-S15). Immunostaining signals from both cortical and hippocampal regions were quantified by investigators blinded to treatment, *APOE* genotype, and sex within paradigm. For morphological analysis of microglia, 8 sub-regions within the CX were imaged at × 40, and the total number of microglia within each frame was classified as type 1 or type 2/3 (Fig. S1B) [43].

### Sequential protein extraction fractions and ELISAs (Aβ, apoE, and IL-1β)

Frozen cortices dissected from the mouse hemi-brains were homogenized using a three-step extraction protocol producing soluble (Tris-buffered saline: TBS), non-ionic detergent (TBS + 1%Triton X-100: TBSX), and insoluble (neutralized formic acid: FA) [44]. Total protein in the TBS and insoluble extracts was quantified using the Bradford assay [44]. Soluble Aβ42, apoE, and interleukin-1 beta (IL-1β) were measured by ELISA following the manufacturer’s instructions, while insoluble Aβ42 and apoE were measured in insoluble fraction (previously described [12, 35, 44, 45]). A list of all the antibodies used is provided in Additional file 2.

### Statistical analysis

GraphPad Prism 9 (for Mac, GraphPad Software, La Jolla, CA) was used for statistical analyses. For all statistical tests, *p* < 0.05 was considered significant. Data was plotted as scatter bar graphs, with the mean and standard error of the mean (SEM). Morris water maze acquisition phase data and body weights were analyzed by repeated measured two- or three-way ANOVA, followed by Tukey’s post hoc tests. All other data were analyzed by unpaired Student’s *t*-test. See Additional file 3 for details on n sizes and statistical comparisons.

## Results

The goal of this study was to evaluate the effect of TLR4 antagonism on neuroinflammation and other markers of AD-relevant pathology in mice that express human *APOE*. To address this goal, we treated EFAD mice from 4 to 6 months of age (Prevention paradigm; PVT) or from 6 to 7 months of age (reversal paradigm; RVS) with either vehicle or IAXO-101 (10 mg/kg ~ 0.3 mg/mouse, 3 subcutaneous injections/week). We used EFAD mice as they overproduce Aβ42 and express human *APOE4* (E4FAD) or *APOE3*. Previous data have demonstrated that E4FAD mice have higher levels of astrogliosis, microgliosis, and Aβ pathology at 6 months of age compared to E3FAD mice [12, 35]. Therefore, in our prevention paradigm, we treated mice from 4 to 6 months or age, and in our reversal paradigm, treatment was from 6 to 7 months. For TLR4 antagonism, we used IAXO-101, as we have previously demonstrated that this compound lowers inflammation in vitro using *APOE4* mixed glial cultures. IAXO-101 has been used in vivo at doses ranging from 0.06 to 0.3 mg/mouse/day via various routes [46–48], and we therefore selected the higher dose for this study (~ 0.3 mg/mouse). There was no difference between the body weights of mice treated with vehicle or IAXO-101 within a specific cohort (Additional file 1: Fig. S7). To evaluate the effects of IAXO-101, we measured neuroinflammation (astrogliosis, microgliosis, glial cell morphology), Aβ pathology, and behavior in EFAD mice.

### PVT paradigm: IAXO-101 lowers neuroinflammation and improves memory in female E4FAD mice

We first focused on whether IAXO-101 could modulate neuroinflammation Aβ pathology and behavior in female E4FAD mice in a prevention paradigm (PVT). Evidence suggests that there is a synergistic effect of *APOE4* and female sex on both AD risk and pathology in humans [49–52] and neuroinflammation in vivo [37, 41, 53–55]. Indeed, female E4FAD mice have higher neuroinflammation and Aβ pathology compared to male E4FAD mice at 6 months of age [15, 19, 56]. Therefore, we considered female E4FAD mice were a logical starting point to test if TLR4 plays a role in regulating neuroinflammation.

**Fig. 1:**
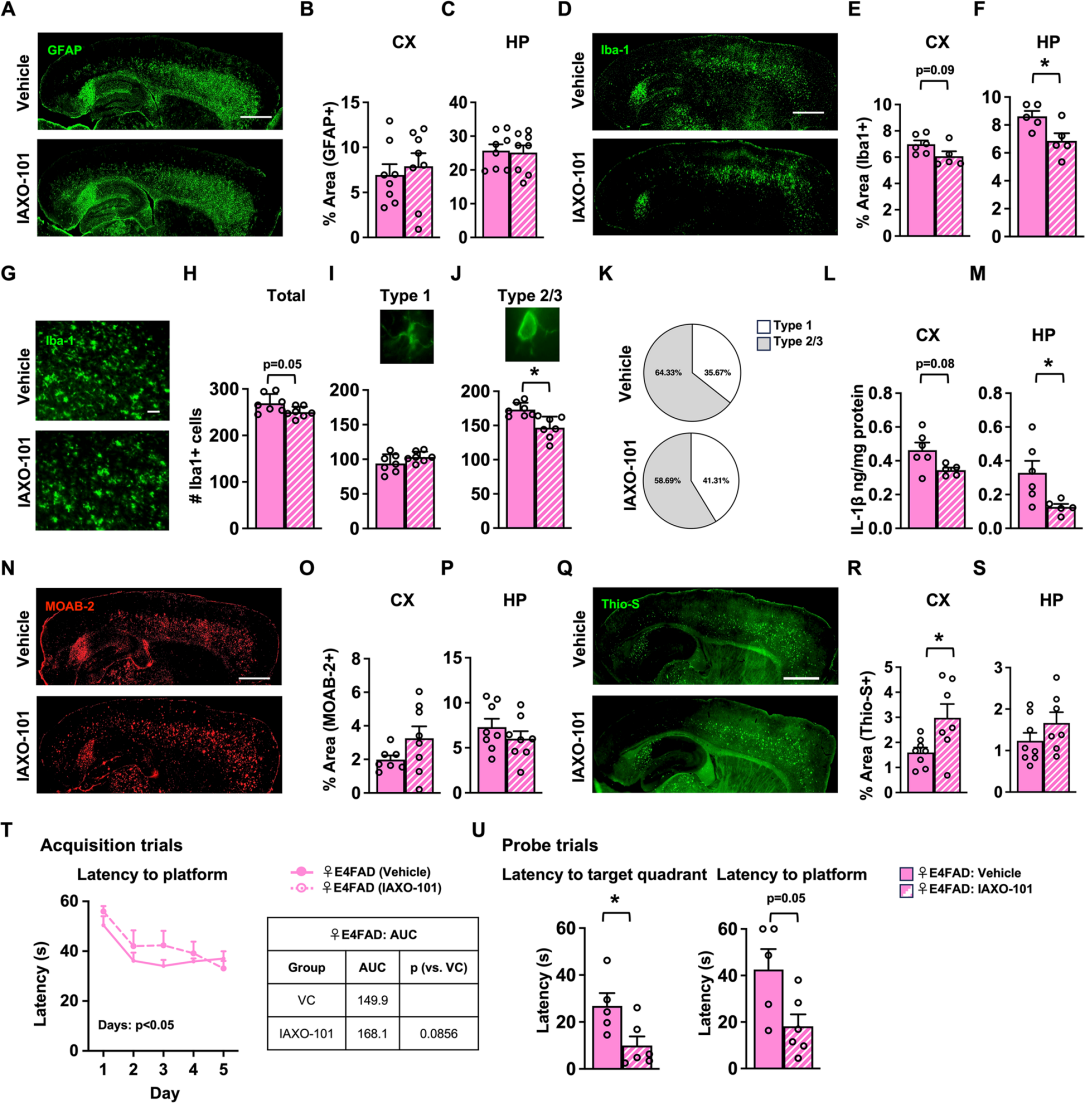
PVT paradigm: IAXO-101 lowers neuroinflammation and improves memory in female E4FAD mice. Female E4FAD mice were treated with IAXO-101 or vehicle from 4 to 6 months of age; PVT paradigm. **A** Representative image of mouse brains immunostained for GFAP (green, scale bars: 1000 μm). Treatment did not impact the percentage of area covered by GFAP in the **B** cortex (CX) [*t*(13.46) = 0.5080, *p* > 0.5] or the **C** hippocampus (HP) [*t*(13.79) = 0.1996, *p* > 0.5]. **D** Microglia Iba-1 coverage (green, scale bars: 1000 μm). IAXO-101 treatment appeared to lower the percentage area covered by Iba-1 in the **E** CX [*t*(7.998) = 1.909, *p* = 0.093] and the **F** HP [*t*(7.259) = 2.608, *p* < 0.05]. **G** Higher power magnification images of Iba-1 in the CX (green, scale bars: 50 μm). IAXO-101 treatment **H** resulted in a lower number of total microglia [*t*(9.809) = 0.5045, *p* = 0.05], and **I** did not affect type 1 microglia; however, **J** resulted in a lower number of type 2/3 microglia. **K** Percentage of type 1 and 2/3 microglia [type 1: *t*(10.81) = 1.456, *p* > 0.1; type 2/3: *t*(10.03) = 0.5080, *p* < 0.05]. Levels of IL-1β were lower with IAXO-101 treatment in the **L** CX [*t*(9) = 2.297, *p* < 0.05] and **M** the HP [*t*(9) = 2.499, *p* < 0.05]. **N** Representative images of Aβ immunostaining (red, scale bars: 1000 μm). There was no effect of IAXO-101 on Aβ levels in the **O** CX [*t*(8.644) = 1.680, *p* > 0.1] and the **P** HP [*t*(13.86) = 1.031, *p* > 0.1]. IAXO-101 treatment increased fibrillar amyloid (**Q** green, scale bars: 1000 μm) in the **R** CX [*t*(6.953) = 2.608, *p* < 0.05] with no effect in the **S** HP [*t*(11.47) = 1.317, *p* > 0.1]. In the Morris water maze test, IAXO-101 treatment **T** had no effect on the learning/acquisition [2-way ANOVA-days: *F*(3.111,40.45) = 11.93, *p* < 0.0001; treatment: *F*(1,13) = 1.011, *p* = 0.333]; however, **U** in the memory/probe trial resulted in lower latency to quadrant and platform [target quadrant: *t*(7.513) = 2.528, *p* < 0.05; platform: *t*(6.604) = 2.388, *p* = 0.05. Data are expressed as mean ± S.E.M. All data analyzed by Student’s *t*-test, except in Morris water maze acquisition trials (two-way repeated measures ANOVA). * *p* < 0.05. See Additional file 3 for *n* sizes and statistical analysis

In the brain, glia (astrocytes and microglia) are key components of the neuroinflammatory response. Alterations in astrocyte reactivity can be evaluated via quantification of GFAP (Fig. 1A–C), S100β [57], and C3 [58] (Fig. S2A-B), which we measured in female E4FAD mice by IHC analysis. We did not find any differences in the levels of any of those astrocytic markers between vehicle and IAXO-101-treated mice. Microglia reactivity can be measured through quantification of Iba-1 (Fig. 1D) and Clec7a [59] levels (IHC). We found that IAXO-101 treatment resulted in a non-significant trend of lower Iba-1 coverage levels in the CX (Fig. 1D–E), and ~ 30% lower levels in the HP (Fig. 1F). There was also a non-significant trend of lower Clec7a coverage in the CX and HP with IAXO-101 treatment (Figure S2C). As cortical Iba-1 was trending lower with IAXO-101, we further evaluated microglial reactivity by calculating the total number in eight sub-regions of the CX (Fig. 1G). We found that total number of microglia (Iba-1+) was lower number with IAXO-101 treatment (Fig. 1H). Microglia states can be classified morphologically as resting (small soma, thin processes; type 1) or reactive (amoeboid; type 2/3), the latter of which is associated with pathological conditions. We found that with IAXO-101 treatment, the number of resting microglia did not change (Fig. 1I), but the number of reactive microglia was lower (Fig. 1J). Further analysis revealed that the distribution of microglial subtypes was altered by IAXO-101 treatment, with a ~ 5% decrease in type 2/3 microglia in IAXO-101 treated mice (Fig. 1K). Changes in microglia reactivity are often associated with altered cytokine levels, including IL-1β. Consistent with lower Iba-1 coverage, IL1-β levels were ~ 25% lower in the CX and ~ 60% lower in the HP of IAXO-101-treated female E4FAD mice (Fig. 1L, M).

One of the proposed functions of glia in AD, particularly microglia, is to clear Aβ [60, 61]. Therefore, Aβ42 levels could have been impacted by IAXO-101 treatment, which we measured using biochemical analysis (BC, ELISA). Surprisingly, there was no effect of IAXO-101 treatment on soluble and insoluble A β42 levels in the CX and HP (BC; Fig. S3A, B). To confirm that IAXO-101 treatment did not modulate extracellular Aβ, we performed immunohistochemical (IHC) analysis (MOAB-2) for Aβ (Fig. 1N) and Thio-S staining for fibrillar amyloid deposits (Fig. 1Q) in female E4FAD mice. IAXO-101 treatment did not impact Aβ deposition (Fig. 1 O, P) or fibrillar amyloid deposits in HP (Fig. 1S) but increased fibrillar amyloid deposits in the CX (Fig. 1R). We also found that soluble and insoluble apoE levels were similar between vehicle- and IAXO-101-treated mice (Fig. S3C, D).

Since IAXO-101 lowered markers of neuroinflammation, we next evaluated the potential impact on learning/memory-relevant using Morris water maze. In the acquisition phase, both groups of mice learned the location of the platform, with no differences between treatments (Fig. 1T). However, in probe trial (memory), IAXO-101-treated female E4FAD mice had lower latency to the target quadrant and platform (Fig. 1U).

In summary, IAXO-101 lowered microglial reactivity and IL-1β levels and improved memory in female E4FAD mice treated in PVT paradigm.

### RVS paradigm: IAXO-101 lowers neuroinflammation and Aβ pathology and improves memory in female E4FAD mice

**Fig. 2:**
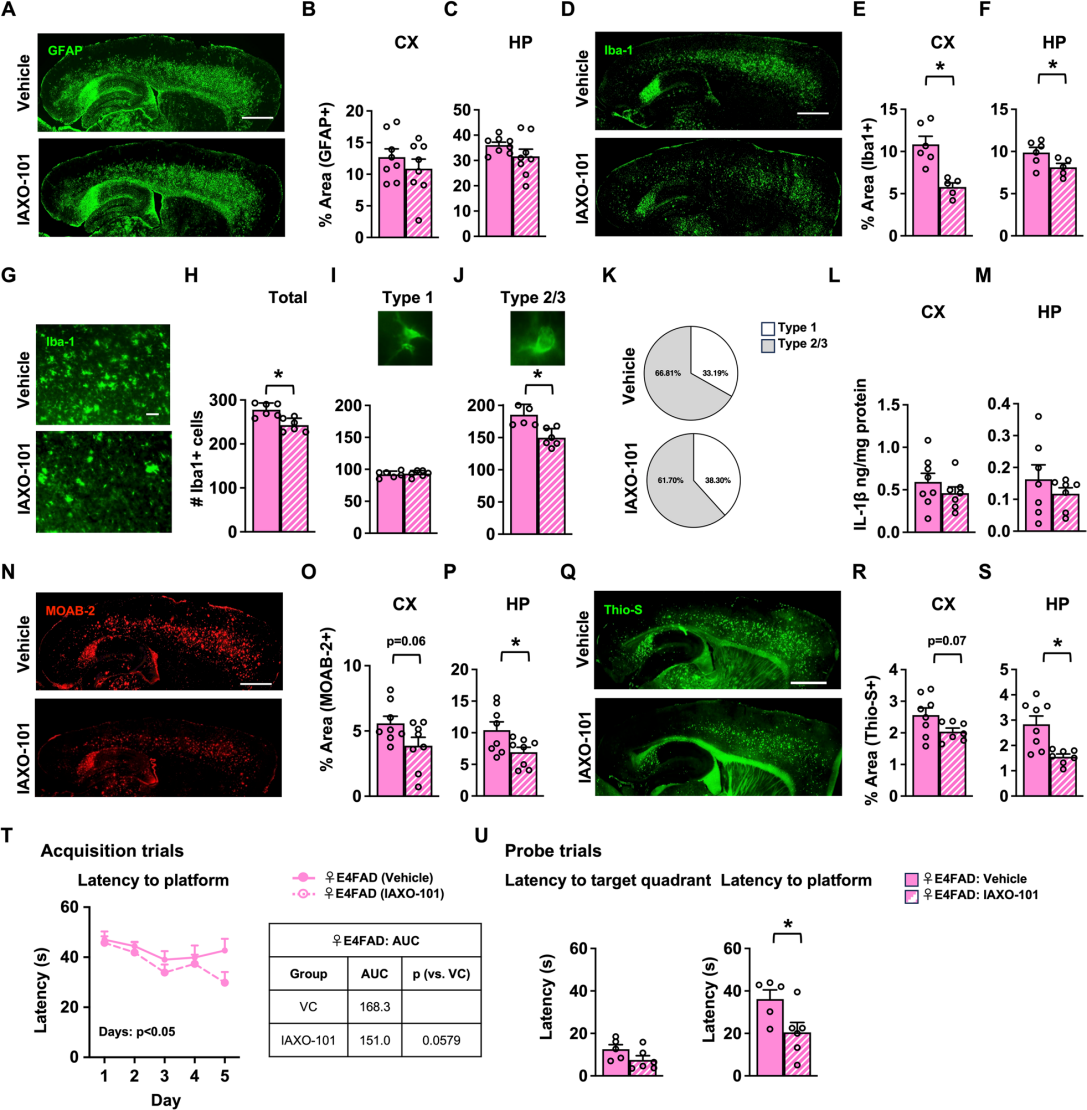
RVS paradigm: IAXO-101 lowers neuroinflammation and Aβ pathology and improves memory in female E4FAD mice. Female E4FAD mice were treated with IAXO-101 or vehicle from 6 to 7 months of age; RVS paradigm. **A** Representative image of mouse brains immunostained for GFAP (green, scale bars: 1000 μm). IAXO-101 treatment did not affect the percentage of area covered by GFAP in the **B** cortex (CX) [*t*(13.71) = 0.9136, *p* > 0.5] or the **C** hippocampus (HP) [*t*(9.278) = 1.448, *p* > 0.5]. **D** Microglia Iba-1 coverage (green, scale bars: 1000 μm). IAXO-101 treatment lowered Iba-1 coverage in the **E** CX [*t*(7.345) = 4.671, *p* < 0.01] and the **F** HP [*t*(8.823) = 2.309, *p* < 0.05]. **G** Higher power magnification images of Iba-1 in the CX (green, scale bars: 50 μm). IAXO-101 treatment **H** resulted in a lower number of total microglia [*t*(9.995) = 3.870, *p* < 0.001], and **I** did not affect type 1 microglia; however, **J** resulted in a lower number of type 2/3 microglia. **K** Percentage of type 1 and 2/3 microglia [type 1: *t*(9.998) = 0.2848, *p* > 0.1; type 2/3: *t*(9.793) = 4.073, *p* < 0.001]. Levels of IL-1β did not change with IAXO-101 treatment in the **L** CX [*t*(12.18) = 1.039, *p* > 0.1] and **M** the HP [*t*(7.979) = 0.928, *p* > 0.1]. **N** Representative images of Aβ immunostaining (red, scale bars: 1000 μm). IAXO-101 treatment lowered Aβ levels in the **O** CX [*t*(13.53) = 2.040, *p* = 0.06] and the **P** HP [*t*(11.46) = 2.294, *p* < 0.05]. IAXO-101 treatment lowered fibrillar amyloid (**Q** green, scale bars: 1000 μm) in the **R** CX [*t*(10.13) = 2.056, *p* = 0.06] and in the **S** HP [*t*(8.520) = 3.725, *p* < 0.01]. In the Morris water maze test, IAXO-101 treatment **T** had no effect on the learning/acquisition [2-way ANOVA-days: *F*(2.907,34.89) = 3.204, *p* < 0.05; treatment: *F*(1,12) = 2.807, *p* = 0.1197]; however, **U** in the memory/probe trial resulted in lower latency to quadrant and platform [target quadrant: *t*(8.663) = 1.7, *p* = 0.1248; platform: *t*(8.991) = 2.462, *p* < 0.05]. Data are expressed as mean ± S.E.M. All data analyzed by Student’s *t*-test, except in Morris water maze acquisition trials *T* (two-way repeated measures ANOVA). * *p* < 0.05. See Additional file 3 for *n* sizes and statistical analysis

An important component of AD research is understanding the extent that targeting a particular function or pathway can modulate pathology and cognition at advanced stages of the disease. Therefore, we next determined the impact of TLR4 antagonism on neuroinflammation and behavior at an age of significant Aβ pathology [19], by treating female E4FAD from 6 to 7 months of age with either vehicle or IAXO-101. Although not significant, GFAP (Fig. 2A–C) and S100β (Fig. S4A) coverage was trending lower with IAXO-101 treatment, with no effect on C3 (Fig. S4B). IAXO-101 lowered Iba-1+ microglia coverage by ~ 40% in the CX (Fig. 2D, E) and ~ 15% in the HP (Fig. 2D, F). Consistent with this observation, we found that IAXO-101 also lowered Clec7a + microglia coverage by 20% in the CX (*p* = 0.09) and HP (Fig. S4C). Subsequent morphological analysis revealed that IAXO-101 treatment was associated with lower number of total number of microglia (Fig. 2H, J), no change in the number of resting microglia (Fig. 2I), and a lower number of reactive microglia (Fig. 2J) and ~ 5% lower distribution of type 2/3 microglia (Fig. 2K). Surprisingly, we did not detect any changes in IL1-β levels in female E4FAD mice (Fig. 2L, M). IAXO-101 treatment also resulted in lower levels of Aβ plaques (Fig. 2N–P) and fibrillar amyloid deposits (Fig. 2Q–S), in both the CX and the HP, but no changes in Aβ (Fig. S3E, F) or apoE levels by BC analysis (Fig. S2G, H). At the behavioral level, IAXO-101 improved memory in the Morris water maze (Fig. 2T, U). Overall, in a RVS paradigm, IAXO-101 treatment lowered neuroinflammation and Aβ pathology and improved memory in female E4FAD mice.

### PVT paradigm: IAXO-101 has no effect on specific neuroinflammatory and behavioral readouts in male E4FAD mice

**Fig. 3:**
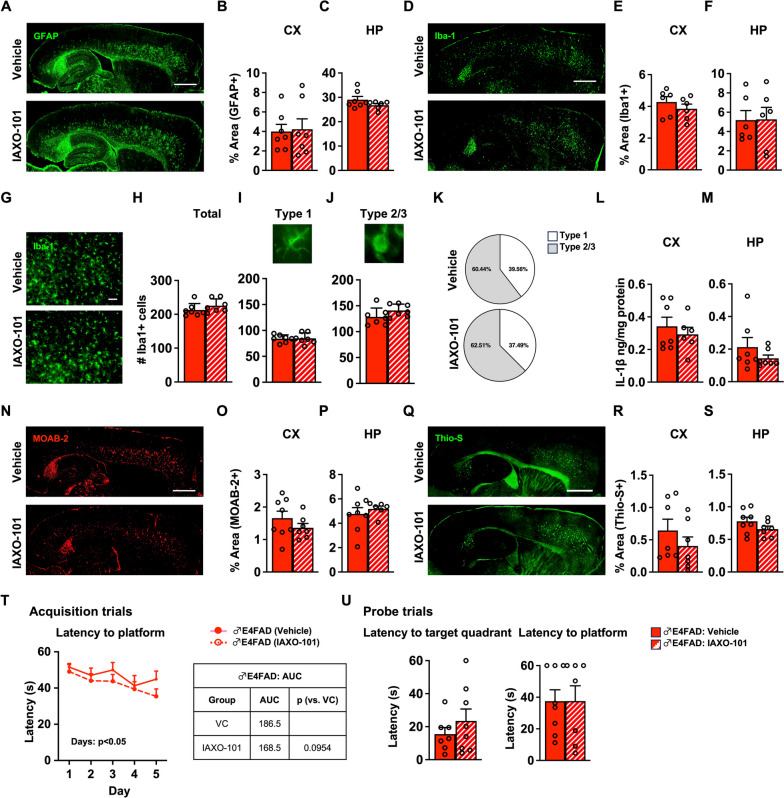
PVT paradigm: IAXO-101 has no effect on specific neuroinflammatory and behavioral readouts in male E4FAD mice. Male E4FAD mice were treated with IAXO-101 or vehicle from 4 to 6 months of age; PVT paradigm. **A** Representative image of mouse brains immunostained for GFAP (green, scale bars: 1000 μm). Treatment did not impact the percentage of area covered by GFAP in the **B** cortex (CX) [*t*(10.68) = 0.1806, *p* > 0.5] or the **C** hippocampus (HP) [*t*(9.084) = 1.503, *p* > 0.1]. **D** Microglia Iba-1 coverage (green, scale bars: 1000 μm). IAXO-101 treatment had no effect on percentage area covered by Iba-1 in the **E** CX [*t*(9.765) = 0.9540, *p* > 0.1] and the **F** HP [*t*(9.829) = 0.3946, *p* > 0.5]. **G** Higher power magnification images of Iba-1 in the CX (green, scale bars: 50 μm). IAXO-101 treatment **H** had no effect on number of total microglia **I** type 1 microglia, **J** type 2/3 microglia, **K** distribution of microglial subtypes [total: *t*(11.98) = 1.226, *p* > 0.1], type 1: *t*(10.73) = 0.09, *p* > 0.5; type 2/3: *t*(10.86) = 1.567, *p* > 0.1] or levels of IL-1β in the **L** CX [*t*(11) = 0.6826, *p* < 0.5] and **M** the HP [*t*(12) = 1.095, *p* > 0.1]. **N** Representative images of Aβ immunostaining (red, scale bars: 1000 μm). There was no effect of IAXO-101 on Aβ levels in the **O** CX [*t*(11.38) = 1.171, *p* > 0.1] and the **P** HP [*t*(9.549) = 0.7235, *p* > 0.1] or in fibrillar amyloid (**Q** green, scale bars: 1000 μm) in the **R** CX [*t*(11.45) = 1.07, *p* > 0.1] and the **S** HP [*t*(11.69) = 1.543, *p* > 0.1]. In the Morris water maze test, IAXO-101 treatment **T** had no effect on the learning/acquisition [2-way ANOVA-days: *F*(3.266,45.72) = 4.532, *p* < 0.01; treatment: *F*(1,14) = 1.056, *p* = 0.322] and **U** in the memory/probe trials [target quadrant: *t*(10.74) = 0.9724, *p* > 0.1; platform: *t*(11.48) = 0.004, *p* > 0.5]. Data are expressed as mean ± S.E.M. All data analyzed by Student’s *t*-test, except in Morris water maze acquisition trials (two-way repeated measures ANOVA). * *p* < 0.05. See Additional file 3 for *n* sizes and statistical analysis

In general, data supports that the combination of female sex and *APOE4* induces a strong neuroinflammatory phenotype in FAD-mice. However, male E4FAD mice also have greater neuroinflammation and Aβ pathology compared to male E3FAD mice at 6 months of age [11, 15, 19, 35]. Therefore, we evaluated whether the beneficial effect of IAXO-101 in lowering neuroinflammation also applied to male E4FAD mice in the same PVT paradigm (4 to 6 months treatment). There was no effect of IAXO-101 treatment on neuroinflammatory markers including GFAP (Fig. 3A–C) and Iba-1 coverage (Fig. 3D–F), total number of microglia (Fig. 3G, H), number of resting (Fig. 3I) and reactive microglia (Fig. 3J), distribution of microglial subtypes (Fig. 3K), and IL1-β levels (Fig. 3L, M). We also did not detect any changes in fibrillar amyloid or Aβ levels (Fig. 3N–S, Fig. S2I, J), apoE levels (Fig. S3K, L), or learning/memory (Fig. 3T, U) after IAXO-101 treatment. Thus, in contrast to female E4FAD mice, IAXO-101 treatment did not result in any beneficial effects on our readouts in male E4FAD mice in a PVT paradigm.

### RVS paradigm: IAXO-101 treatment lowered Iba-1 coverage but had no effect on Aβ pathology and behavior in male E4FAD mice

**Fig. 4:**
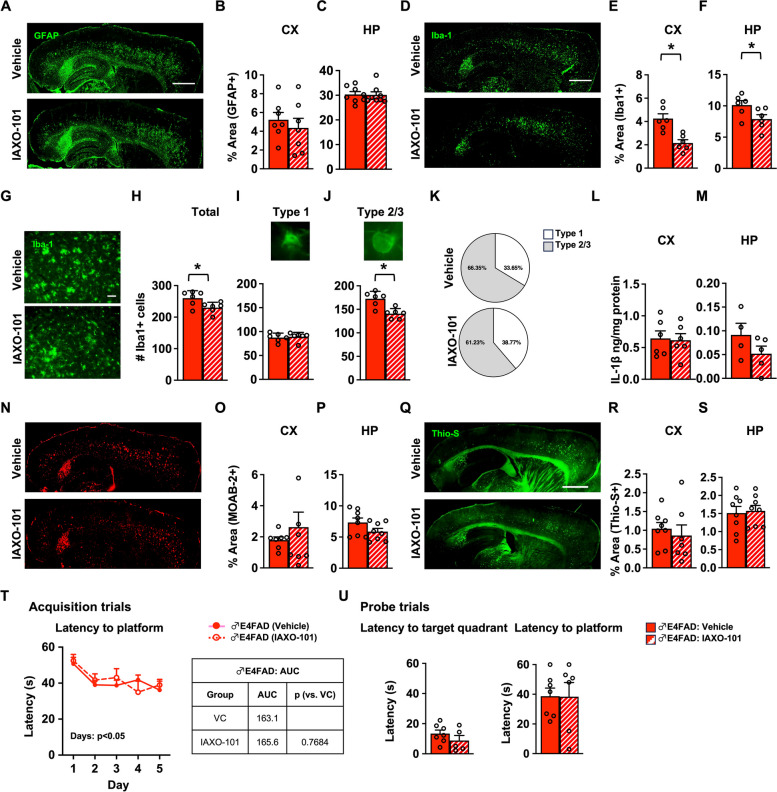
RVS paradigm: IAXO-101 treatment lowered Iba-1 coverage but had no effect on Aβ pathology and behavior in male E4FAD mice. Male E4FAD mice were treated with IAXO-101 or vehicle from 6 to 7 months of age; RVS paradigm. **A** Representative image of mouse brains immunostained for GFAP (green, scale bars: 1000 μm). Treatment did not impact the percentage of area covered by GFAP in the **B** cortex (CX) [*t*(11.16) = 0.6788, *p* > 0.5] or the **C** hippocampus (HP) [*t*(13.87) = 0.1201, *p* > 0.5]. **D** Microglia Iba-1 coverage (green, scale bars: 1000 μm). IAXO-101 treatment lowered percentage area covered by Iba-1 in the **E** CX [*t*(8.742) = 4.186, *p* < 0.05] and the **F** HP [*t*(9.993) = 2.253, *p* < 0.5]. **G** Higher power magnification images of Iba-1 in the CX (green, scale bars: 50 μm). IAXO-101 treatment **H** lowered number of total microglia **I** but did not affect type 1 microglia [total: *t*(9.035) = 2.497, *p* < 0.05; type 1: *t*(10.00) = 0.3145, *p* > 0.5]. However, IAXO-101 treatment lowered **J** type 2/3 microglia [type 2/3: *t*(8.845) = 4.032, *p* > 0.1] and modified the **K** distribution of microglial subtypes. Surprisingly, there was no effect of IAXO-101 on the levels of IL-1β in the **L** CX [*t*(9.835) = 0.177, *p* > 0.5] and **M** the HP [*t*(5.356) = 1.348 *p* > 0.1]. **N** Representative images of Aβ immunostaining (red, scale bars: 1000 μm). There was no effect of IAXO-101 on Aβ levels in the **O** CX [*t*(7.636) = 0.8443, *p* > 0.1] and the **P** HP [*t*(12.63) = 1.643, *p* > 0.1] or in fibrillar amyloid (**Q** green, scale bars: 1000 μm) in the **R** CX [*t*(9.761) = 10.558, *p* > 0.5] and the **S** HP [*t*(13.47) = 0.2563, *p* > 0.5]. In the Morris water maze test, IAXO-101 treatment **T** had no effect on the learning/acquisition [2-way ANOVA-days: *F*(2.774,38.84) = 6.170, *p* < 0.01; treatment: *F*(1,14) = 0.064, *p* > 0.5] and **U** in the memory/probe trials [target quadrant: *t*(8.075) = 0.035, *p* > 0.5; platform *t*(7.641) = 1.134, *p* > 0.1]. Data are expressed as mean ± S.E.M. All data analyzed by Student’s *t*-test, except in Morris water maze acquisition trials (two-way repeated measures ANOVA). * *p* < 0.05. See Additional file 3 for *n* sizes and statistical analysis

One possible explanation for a lack of an effect of IAXO-101 in PVT paradigms in male E4FAD mice is that at early stages/ages of pathology the contribution of TLR4 to neuroinflammation and/or behavioral function is minimal. To explore this concept, we next evaluated the effect of TLR4 antagonism on behavior in male E4FAD mice at ages of more advanced Aβ levels and higher neuroinflammation. There are age-dependent increases in Aβ pathology and neuroinflammation in male E4FAD mice from 4 to 6 months of age [35]. Therefore, we next determined whether IAXO-101 treatment from 6 to 7 months of age was beneficial in male E4FAD mice. IAX0-101 did not affect GFAP coverage (Fig. 4A–C). However, IAXO-101 treatment reduced Iba-1 coverage by ~ 50% the CX (Fig. 4D, E) and ~ 25% in the HP (Fig. 4D, F). In addition, compared to vehicle, IAXO-101 treatment resulted in a lower number of total microglia (Fig. 4G, H) and lower percentage of reactive microglial subtypes (Fig. 4I–K) without modulating IL-1β levels (Fig. 4L, M). There was no effect of IAXO-101 on Aβ levels (Fig. S3O, P, Fig. 4N-S), apoE levels (Fig. S3M, N), or learning and memory (Fig. 4T, U). Thus, in general, the effect of IAXO-101 in male E4FAD mice may be limited to lowering neuroinflammatory markers without affecting Aβ pathology or cognition.

### PVT/RVS paradigm: IAXO-101 had no effect in female E3FAD mice

Our data suggested that IAXO-101 is more beneficial in female E4FAD mice than males, particularly for microglia, which raised the question of whether TLR4 antagonism would be beneficial for female *APOE3* carriers. Indeed, neuroinflammation, including microglial number and morphology, has been shown to be higher in females in rodents [62] and humans [63]. Therefore, we tested if IAXO-101 affected the levels of neuroinflammation in female E3FAD mice in both PVT and RVS paradigms. We found that IAXO-101 did not affect neuroinflammation (PVT: Fig. S5A-F; RVS: Fig. S6A-F), Aβ or apoE levels (PVT: Fig. S5G-J; RVS: Fig. S6G-J) or learning and memory (PVT: Fig. S5K, L; RVS: Fig. S6K, L) in female E3FAD mice. Therefore, unlike female *APOE4* mice, IAXO-101 was not effective at mitigating pathology and behavior in female E3FAD mice.

## Discussion

Neuroinflammation mediated by microglia is increasingly recognized as an important component of AD pathogenesis. However, the role of microglia function in AD is complex and is dependent on many factors including activation state and disease stage. For example, it has been proposed that moderately activated microglia are neuroprotective in the early stages of AD, while chronically activated microglia may be detrimental in later stages (reviewed in [64–66]). Genetic AD risk factors and sex may also impact the extent that microglia are beneficial, neutral, or detrimental for cognitive function in AD. Our study supports that reducing microglial neuroinflammation is particularly or specifically beneficial for improving memory in female *APOE4* carriers. Although there are limitations (see below), the implication is that neuroinflammation may contribute to higher risk and progression of AD found in *APOE4* females. Consistent with this concept, human data demonstrate that compared to *APOE3*, female *APOE4* AD patients have earlier onset of cognitive deficits (reviewed in [67]) and increased neuroinflammation, including increased activation of microglia and secretion of cytokines [7–10]. Similarly, in mice, greater impairment in learning/memory and neuroinflammation is seen in females compared to males in multiple FAD mouse models [68–72], with *APOE4* exacerbating these effects [11, 15, 19]. Thus, collectively, the combination of female sex and *APOE4* may produce an environment that specifically results in greater microglial inflammatory respond that leads to greater cognitive dysfunction in AD.

The concept that *APOE4* and female sex synergistically impact neuroinflammation to impair cognition raises the important discussion of the underlying mechanisms. In general, apoE4 has been proposed to alter cellular function in different ways [73]. At the structural level, apoE isoforms differ by two amino acids at position 112 and 158, which affects protein folding and is thought to result in changes to lipidation (apoE4 < apoE3) stability and levels (apoE4 < apoE3) [74, 75]. These structural changes are proposed to induce differences apoE availability, distribution, aggregation, receptor binding affinities and signaling that modulates peripheral inflammation (via macrophages and other immune cells), cerebrovascular function (via endothelial cells, pericytes and astrocytes), Aβ levels, lipid trafficking, and neuron function directly (reviewed in [3, 76–79]). Therefore, the impact of *APOE* on other cell types could indirectly impact glial morphology/activation. In addition, apoE can be produced by microglia to impact their activity. For example, microglia expressing *APOE4* has altered immune responses and metabolism in vitro [18, 80, 81], and selective ablation of microglial apoE4 in a tauopathy mouse model blocks brain atrophy [82, 83]. Thus, collectively *APOE4* could indirectly and directly results in higher potential for microglia reactivity to be exacerbated by female sex.

The underlying mechanisms of how female sex impacts AD progression is unclear. One proposed mechanism is that loss of sex hormones at menopause results in greater AD susceptibility. Indeed, ovariectomy results in behavioral impairments and accelerated aging [84–86] in women as well as in rodents [87, 88]. Importantly, ovariectomy results in greater neuroinflammation in rodents including microglial activation [87–91]. Therefore, as estrogen demonstrates anti-inflammatory activity via the estrogen receptors, the loss of estrogen may promote a microglial response [92]. In addition to direct effects on microglia, the loss of estrogen also modulates AD pathology, as in general, Aβ deposition is increased in women during the menopausal transition [93, 94], in post-mortem brains of ovariectomized women [95], and in brains of ovariectomized rodents with some conflicting results in FAD mice (discussed in [96]). Thus, menopause could indirectly and directly modulate microglial activation in a way that is particularly pronounced with *APOE4* carriers. However, in our study, mice were not ovariectomized, suggesting a different mechanism of action for how female sex and *APOE4* impacted glial activation. In general, there are sex differences in microglial numbers, function, and response to an acute/chronic insult and age without menopause mimics [62, 97], including in *APOE4* FAD mice [11, 34]. Thus, one potential explanation is with *APOE4*, there is greater age-dependent changes in sex hormones or their receptors that influence the inflammatory state of microglia, thereby modulating its function. Alternatively, the difference between sex hormones and immune-related genes on the X chromosome may result in higher age-related neuroinflammation (reviewed in [98–101]). Future studies could focus on understanding in more detail the precise mechanism(s) though which sex and *APOE4* increase neuroinflammation and microglial activation.

Our data supports a specific role of TLR4 in modulating neuroinflammation and cognition in *APOE4* females. Data from human, in vivo and mouse studies have led to the idea that blocking TLR4 is a potential AD therapeutic strategy albeit in a non-*APOE4* context. Several single nucleotide polymorphisms (SNPs) in *TLR4* have been associated with modulating AD risk in humans. For example, the G coding variant of rs4986790(A/G) [31, 32] and the C variant of rs11367(G/C) in TLR4 is associated with decreased AD risk [102]. Consistent with a detrimental role of TLR4 in AD, in vitro and ex vivo studies supports that these variants reduce TLR4 activation, resulting in attenuation of proinflammatory signaling [103, 104]. Several minor alleles of TLR4 SNPs (rs10759930, rs1927914, rs1927911, rs12377632) were also found to increase the risk of AD [33], although their function is yet to be understood. Further evidence for a role of TLR4 in AD include that levels of LPS [105] and TLR4 expression are increased in the brains of AD patients [28, 29] and FAD mice [29, 30]. In addition, Aβ has been shown to activate TLR4 resulting in reactive oxygen species production in microglia in vitro [106], and deletion of the TLR4 co-receptor CD14 resulted in lower amyloid plaques in vivo [107]. An important question for further study is whether SNPs in TLR4, TLR4 expression data, and in vitro functional data are modulated by *APOE* and sex in AD-relevant context. In terms of *APOE* alone, LPS (TLR4 agonist) induces *APOE*-specific neuroinflammatory markers in multiple tissues (*APOE4* > *APOE3*) in vivo [14, 108], ex vivo [108], and in vitro [109] with an inconsistent sex effect [110]. In terms of female sex, TLR4 expression is greater in multiple tissues in females than males in human and rodents [110], and the estrogen receptor may impact TLR signaling [92]. Potentially, therefore, the combination of *APOE* and female sex may specifically result in altered TLR4-mediated neuroinflammation to impact cognition.

The implication of our findings is that targeting neuroinflammation is a potential AD therapeutic approach; for certain high-risk groups, however, current human studies are conflicted. To date, most data is derived from epidemiological studies, and a few clinical trials focused on established drugs with known anti-inflammatory properties. One class is NSAIDs that inhibit COX1 and 2 activities. Epidemiological and in vivo studies indicated that non-steroidal anti-inflammatory drugs (NSAIDs) lower AD risk and pathology (reviewed in [111, 112]). However, NSAIDs have not been found efficacious in some AD clinical trials [20]. In addition, a recent re-analysis of the NSAID epidemiological data in an ADNI dataset revealed that although some NSAIDs are associated with decreased AD prevalence, they did not affect cognitive decline in AD patients [21]. A second drug used to evaluate neuroinflammation as an AD target is paracetamol that has unknown and likely pleiotropic mechanisms of action. For example, paracetamol is used for its anti-pyretic, anti-inflammatory, and analgesic actions that may be mediated through lowering prostaglandin levels, inhibiting COX, altering cannabinoid signaling, and also blocking neuronal sodium channels [113]. Paracetamol has been shown to lower AD prevalence; however, whether the beneficial effects of paracetamol in AD are due to effects on inflammation or other mechanisms is unclear [21]. In addition to further understanding the mechanism of action of anti-inflammatories, there are several important considerations for clinically targeting neuroinflammation in AD. One is that neuroinflammation is complex and recognition of pathogens or danger-associated molecules occurs in multiple cell types, through a large array of receptors that produce an equally complex signaling and functional response. Thus, targeted one specific pathway may not alone be sufficient to improve all aspects of neuroinflammation in AD. Therefore, a detailed understanding of how different pathways contribute to any proposed detrimental neuroinflammatory phenotype in AD, including which aspects are more proximal to neuronal dysfunction, is important for interpreting clinical trial data. A second is that whether an inflammatory mediator or receptor is beneficial or detrimental for AD progression may depend on the stage of disease. For example, it has proposed that anti-inflammatories may be efficacious as a prophylactic treatment for *APOE4* carriers to prevent AD [21]. A third, as highlighted by our study, is that the contribution of neuroinflammation to neural dysfunction may be dependent on risk factors (e.g., sex, *APOE* genotype, lifestyle, other genetic or environmental factors). Perhaps future studies in larger heterogenous populations could provide an explanation for determining whether NSAIDs have a greater impact in specific groups.

## Limitations

There are limitations in the extent that we can conclude TLR4 and/or neuroinflammation specifically contributes to neural dysfunction in female *APOE4* mice. In fact, studies have shown that microglial activation and neuroinflammation are greater with *APOE4* than *APOE3* in males using *APOE* knock in mice [14, 114–116], FAD mice [11, 15, 43], and AD patients [117, 118]. Indeed, it is important to note that we found that IAXO-101 lowered microglial number and reactivity in older male E4FAD mice, although performance in the Morris water maze test was not affected. The contribution of TLR4 to microglial activation maybe dependent on age and/or Aβ pathology, which is why IAXO-101 modulated microglia number/reactivity when treatment occurred in older rather than younger male E4FAD mice. The lack of a behavioral benefit in male E4FAD mice as opposed to female E4FAD mice with IAXO-101 treatment could be due multifactorial factors. For example, there could be the same age-dependent consideration as for microglial activation within males, such that beneficial effects in behavior would have been observed if treatment had continued for longer. Alternatively, the contribution of microglia to performance in Morris water maze performance may be greater in females than males. Furthermore, a more comprehensive set of behavioral tests along with expanded markers of neuron function may have revealed that IAXO-101 was beneficial in E4FAD male mice. In addition, *APOE3*-FAD also shows age-dependent increases in glial activation and cognitive impairment [19, 40]. Therefore, future studies could explore if TLR4, neuroinflammation, and/or microglia activity are more proximal to behavioral dysfunction in older male E4FAD, female E3FAD, and male E3FAD mice. Such studies could reveal if there are different optimal treatment windows for targeting neuroinflammation within each *APOE* genotype and sex combination or support specificity for female *APOE4* carriers.

Our study was a proof-of-concept design centered on TLR4 antagonism. However, TLR4 may not be the main contribution to inflammation for all *APOE* genotypes and sexes. For example, there is the possibility that TLR4 activation is more pronounced in female *APOE4* mice, but other inflammatory pathways are important for *APOE4* male mice or *APOE3* mice. Indeed, sex hormones and X chromosome genes can differentially regulate TLR4 [98, 99, 119] and testosterone decreases expression and function of pro-inflammatory cytokines [120]. In addition, as mentioned above, different inflammatory pathways may be important at different ages/stages of pathology. Therefore, identifying how *APOE* genotype, sex, and age impact the neuroinflammatory phenotype, including specific pathways, is important for ultimately advancing our mechanistic understanding of neuroinflammation in AD.

There were also experimental limitations surrounding the way that we targeted TLR4. One aspect is the dose of IAXO-101, which we selected based on previous publications; however, we did not conduct detailed PK studies to determine brain and plasma levels. Therefore, IAXO-101 levels in the plasma and brain may have been different for each group of mice, such as higher in female E4FAD mice, which may explain the beneficial effects. In addition, IAXO-101 inhibits TLR4 activation by two mechanisms: sequestering LPS by forming stable co-aggregates or competing with LPS for binding to CD14 and myeloid differentiation factor 2 [121]. This mechanism of action is based on LPS; however, it is unclear what pathways lead to TLR4 activation in female E4FAD mice. Thus, another approach would be to use competitive inhibitors of TLR4 and/inhibitors of TLR4 signaling down-stream. There is also the question of whether there are cell type specific functions of TLR4. TLR4 is expressed by virtually every cell in the body that could have opposed or synergistic functions. In fact, both activating and inhibiting TLR4 has been shown to decrease AD-related pathology in FAD mice with opposing effects on neuroinflammation [122, 123]. Therefore, evaluating the cell type-specific functions of TLR4 in EFAD mice using detailed mechanistic readouts for neuroinflammation, neuron function, and behavior as well as plasma biomarkers is important for fully evaluating TLR4 as a therapeutic target in AD.

## Conclusion

Our study demonstrates that the TLR4 antagonist IAXO-101 improves memory and reduced neuroinflammation with minimal effects of Aβ pathology in female *APOE4* mice. Thus, TLR4 inhibition is a potential therapeutic approach for AD, particularly in female *APOE4* carriers. In addition, these results support stratification of preclinical and clinical studies that target neuroinflammation by *APOE*, sex, and treatment window.

### Supplementary Information

**Additional file 1: Supplementary figures.**

**Additional file 2:** **Table 1.** List of reagents and antibodies.

**Additional file 3. **Raw data and statistical analysis results presented in the manuscript.

## Data Availability

The datasets used and/or analyzed during the current study are available from the corresponding author on reasonable request.

## Abbreviations

Aβ: Amyloid beta
AD: Alzheimer’s disease
Apolipoprotein E: ApoE (gene: *APOE*)
CD14: Cluster of differentiation 14
COX: Cyclooxygenase
CX: Cortex
FAD: Familial Alzheimer’s disease
IL: Interleukin
LPS: Lipopolysaccharide
NSAID: Non-steroidal anti-inflammatory drugs
PVT: Prevention
RVS: Reversal
SNP: Single nucleotide polymorphism
TLR: Toll-like receptor
