# Supplementary material for: A small-molecule TLR4 antagonist reduced neuroinflammation in female E4FAD mice
Source: Alzheimers Res Ther. 2023 Oct 19;15:181. doi: 10.1186/s13195-023-01330-6 (PMC10585767; doi:10.1186/s13195-023-01330-6)
Supplement: Supplementary file 1 — Additional file 1: Supplementary figures. [file 13195_2023_1330_MOESM1_ESM.docx]

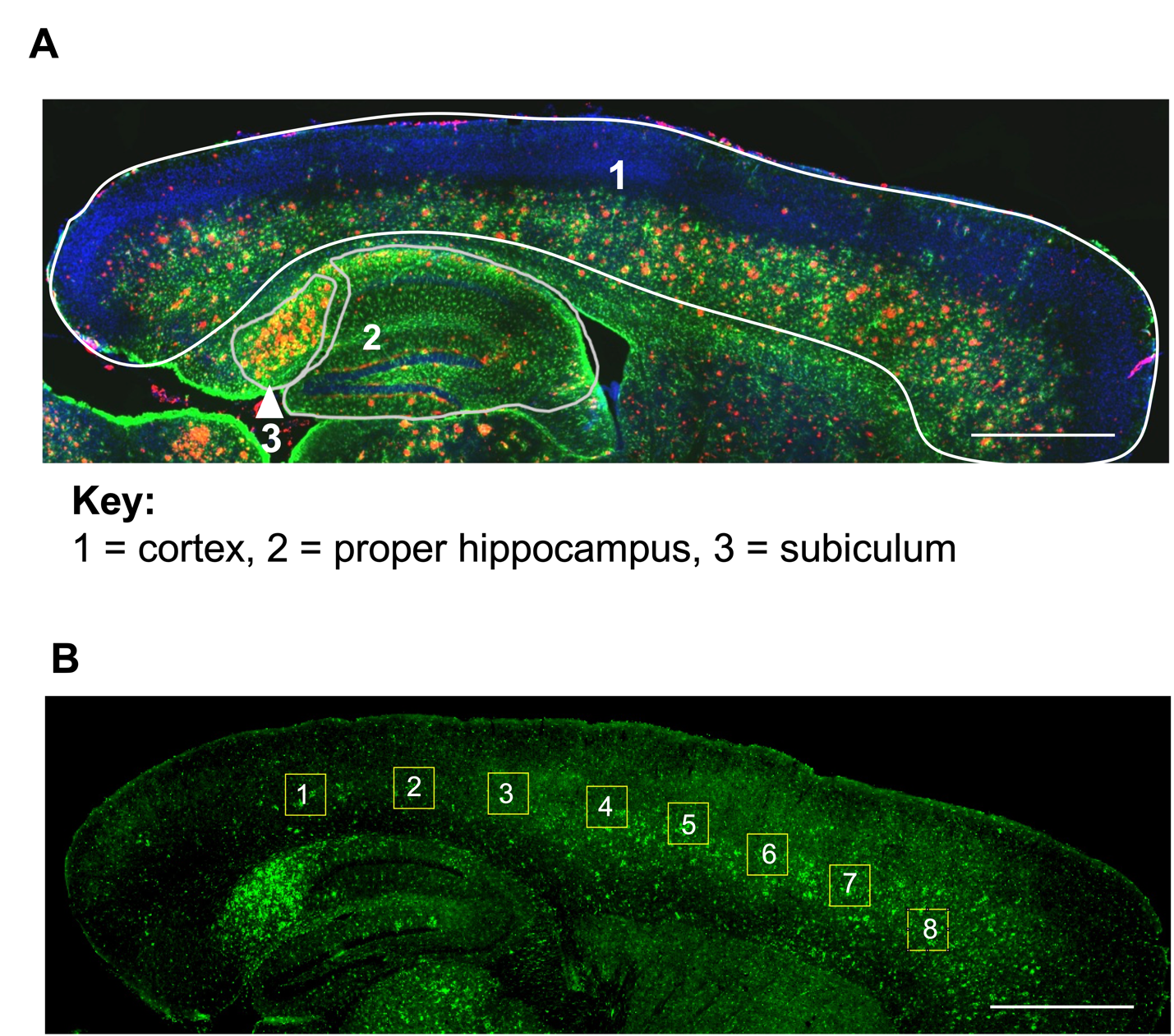


**Fig. S1** **A** Representative image of cortical and hippocampal brain regions used to quantify the percentage of area covered by Aβ, GFAP, Iba-1 and Thio-S. **B** Microglia numbers and morphologically were quantified in specific sub-regions of the cortex.

**Fig. S2 PVT paradigm: IAXO-101 had no effect on astrocytic markers but trended to lower reactive microglial markers in female E4FAD mice.** Female E4FAD mice were treated with IAXO-101 or vehicle from 4 to 6 months of age in the PVT paradigm. IAXO-101 treatment did not affect percentage of area covered by S100β in the CX [t(9.028)= 0.0379, *p*>0.5] and the HP [t(9.914)= 0.1243, *p*>0.5] (**A**) (S100β immunostained brain sections, Green, scale bars: 1000µm). There was also no effect of IAXO-101 on C3 coverage in CX [t(9.989)= 0.3803, *p*>0.5] and HP [t(6.742)= 0.3510, *p*>0.5] (C3 immunostained brain sections, Red, scale bars: 1000µm) (**B**). There was a non-significant trend of lower Clec7a coverage in the CX and HP with IAXO-101 treatment in CX [t(7.087)= 0.2554, *p*>0.5] and HP [t(9.188)= 1.860, *p*=0.095] (Clec7a immunostained brain sections, Green, scale bars: 1000µm) (**C**). Data are expressed as mean +/- S.E.M. All data were analyzed by Student’s t-test. **p*<0.05. See Additional file 3 for details on n sizes and statistical analysis.


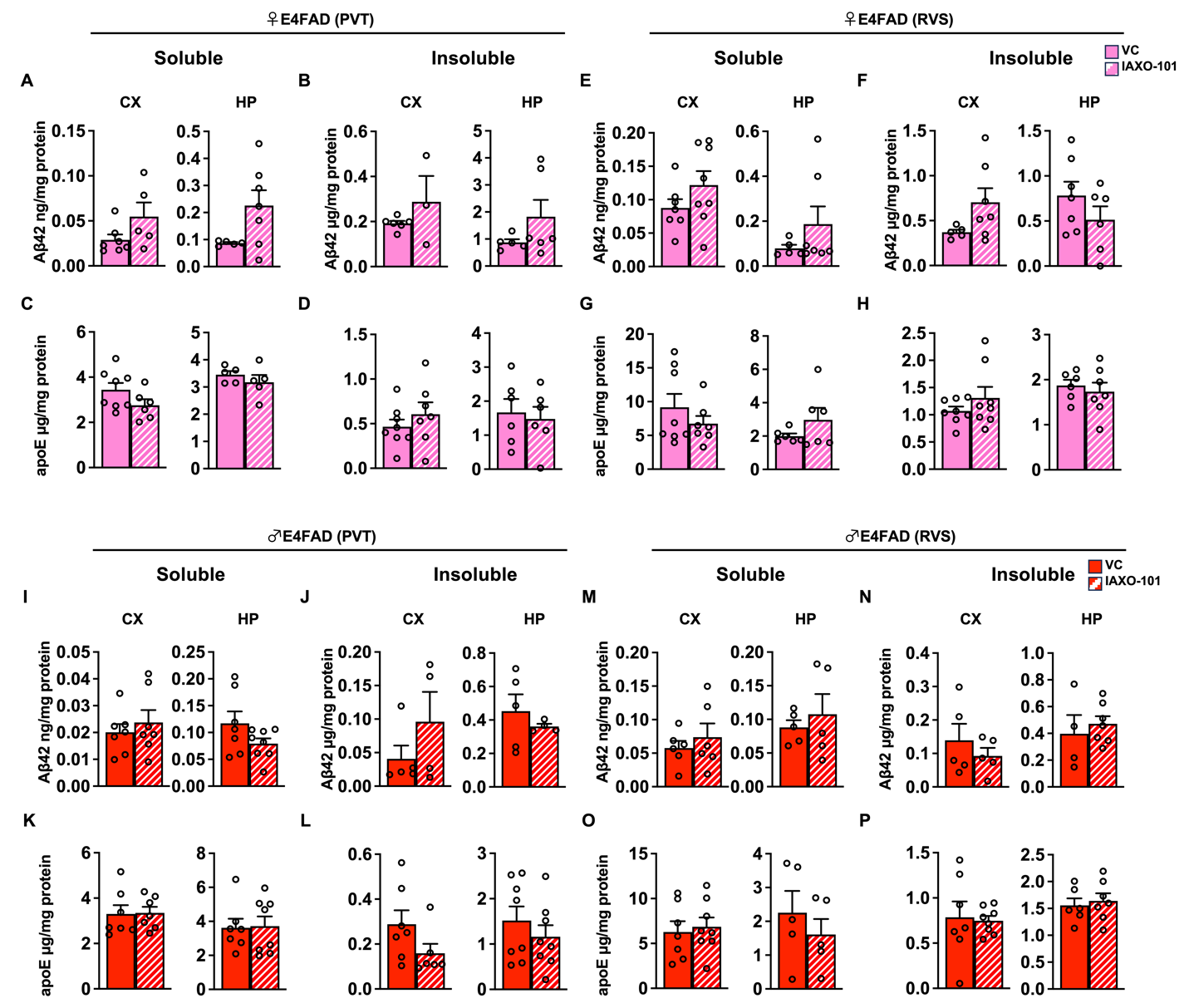


**Fig. S3 PVT/RVS paradigm: IAXO-101 treatment had no effect on Aβ and apoE levels in males or female E4FAD mice.** Soluble and insoluble levels of Aβ and apoE in cortex (CX) and hippocampus (HP) of female and male E4FAD mice were measured by ELISAs. **A-D**; Female E4FAD PVT paradigm. **E-H** Female E4FAD RVS paradigm. **I-L**; Male E4FAD PVT paradigm. **M-P**; Male E4FAD RVS paradigm. Data are expressed as mean +/- S.E.M. All data were analyzed by Student’s t-test. **p*<0.05. See Additional file 3 for details on n sizes and statistical analysis.

**Fig. S4 RVS paradigm: IAXO-101 had trending effects on lowering select glial markers in female E4FAD mice.** Female E4FAD mice were treated with IAXO-101 or vehicle from 6 to 7 months of age in the RVS paradigm. S100β (Fig. S15B) coverage was trending lower with IAXO-101 treatment in the CX [t(7.072)=1.787, *p*=0.12] and the HP [t t(9.980)= 0.2175, *p*>0.5] (**A**) (S100β immunostained brain sections, Green, scale bars: 1000µm). There was no effect of IAXO-101 on C3 coverage in CX [t(9.360)= 0.8366, *p*>0.1] and HP [t(9.587)= 0.5310, *p*>0.5] (C3 immunostained brain sections, Red, scale bars: 1000µm) (**B**). IAXO-101 lowered Clec7a in the CX [t(7.620)= 1.919, *p*=0.0931] and HP [t(7.611)= 2.562, *p*<0.5] (Clec7a immunostained brain sections, Green, scale bars: 1000µm) (**C**). Data are expressed as mean +/- S.E.M. All data were analyzed by Student’s t-test. **p*<0.05. See Additional file 3 for details on n sizes and statistical analysis.

**Fig. S5 PVT paradigm: IAXO-101 had no effect in female E3FAD mice.** Female E3FAD mice were treated with IAXO-101 or vehicle from 4 to 6 months of age in the PVT paradigm. IAXO-101 treatment did not affect percentage of area covered by GFAP in the (**A**) CX [t(6.194)= 0.1322, *p*>0.5] and the (**B**) HP [t(5.024)= 0.1322, *p*>0.1] (GFAP immunostained brain sections, Green, scale bars: 1000µm) and Iba-1 coverage in the (**C)** CX [t(9.621)= 1.540, *p*>0.1] and the (**D**) HP [t(7.368)= 1.997, *p*>0.05] (Iba-1 immunostained brain sections, Green, scale bars: 1000µm). There was also no effect of IAXO-101 on IL-1β in the (**E**) CX [t(11)= 0.7075, *p*>0.5] and (**F**) the HP CX [t(11)= 1.917, *p*>0.5]. IAXO-101 had no effect of Aβ levels in the (**G**) CX [t(9.994)= 0.4377, *p*>0.5] and in the (**H**) HP [t(6.401)= 1.975, *p*>0.05] and fibrillar amyloid levels in the (**I**) CX [t(7.964)= 1.866, *p*>0.05] and in the (**J**) HP [t(6.204)= 1.192, *p*>0.05]. In the Morris water maze test, IAXO-101 treatment (**K)** had no effect on the learning/Acquisition [2-way ANOVA-Days: F (2.730, 38.22)=7.101, *p*<0.001; Treatment: F(1,14)=0.1374, *p*=007164], and (**L)** in the memory/probe trials [Target quadrant: t(10.38)=1.047, *p*>0.1; Platform: t(13.76)=0.086, *p*>0.5]. Data are expressed as mean +/- S.E.M. All data analyzed by Student’s t-test, except in M (two-way ANOVA). * *p*<0.05. See Additional file 3 for *n* sizes and statistical analysis.

**Fig. S6 RVS paradigm: IAXO-101 had no effect in female E3FAD mice.** Female E3FAD mice were treated with IAXO-101 or vehicle from 6 to 7 months of age in the RVS paradigm. IAXO-101 treatment did not affect percentage of area covered by GFAP in the (**A**) CX [t(13.32)= 0.038, p>0.5] and the (**B**) HP [t(9.632)= 1.019, p>0.5] (GFAP immunostained brain sections, Green, scale bars: 1000µm) and Iba-1 coverage in the (**C**) CX [t(7.416)=2.323, p=0.05] and the (**D**) HP [t(5.993)= 1.281, p>0.1] (Iba-1 immunostained brain sections, Green, scale bars: 1000µm). There was also no effect of IAXO-101 on IL-1β in the (**E**) CX [t(9.509)=0.8827, p>0.1] and (**F**) the HP CX [t(6.972)= 0.8594, p>0.1]. IAXO-101 had no effect of Aβ levels in the (**G**) CX [t(13.29)=0.986, p>0.1] and in the (**H**) HP [t(11.20)=1.34, p>0.1] and fibrillar amyloid levels in the (**I**) CX [t(9.05)=1.088, p>0.1] and in the (**J**) HP [t(11.88)=0.3063, p>0.5]. In the Morris water maze test, IAXO-101 treatment (**K**) had no effect on the learning/Acquisition [2-way ANOVA-Days: F(3.191,44.67)=7.492, p<0.001; Treatment: F(1,14)=0.2545, p>0.5], and (**L**) in the memory/probe trials [Target quadrant: t(5.839)=0.6491, p>0.5; Platform: t(7.444)= 0.203, p>0.5]. Data are expressed as mean +/- S.E.M. All data analyzed by Student’s t-test, except in M (two-way ANOVA). * p<0.05. See Additional file 3 for n sizes and statistical analysis.

**Fig. S7** IAXO did not affect body weights of during the treatment in both (**A**) PVT and (**B**) RVS paradigms in female E4FAD, male E4FAD and female E3FAD mice. Data are expressed as mean +/- S.E.M. and analyzed by 2-way ANOVA for week and treatment effect within *APOE* genotype and sex combination. See Additional file 3 for details on n sizes and statistical analysis.

**Fig. S8** PVT paradigm: Representative close-up images of GFAP immunostaining (Green, scale bar: 500µm) in the (**A**) CX and the (**B**) HP female E4FAD, male E4FAD, and female E3FAD mice treated in PVT paradigm. Images taken at 20x magnification.

**Fig. S9** PVT paradigm: Representative close-up images of Iba-1 immunostaining (Green, scale bar: 500µm) in the (**A**) CX and the (**B**) HP female E4FAD, male E4FAD, and female E3FAD mice treated in PVT paradigm. Images taken at 20x magnification.

**Fig. S10** PVT paradigm: Representative close-up images of MOAB-2 immunostaining (Red, scale bar: 500µm) in the (**A**) CX and the (**B**) HP female E4FAD, male E4FAD, and female E3FAD mice treated in PVT paradigm. Images taken at 20x magnification.

**Fig. S11** PVT paradigm: Representative close-up images of Thio-S immunostaining (Green, scale bar: 500µm) in the (**A**) CX and the (**B**) HP female E4FAD, male E4FAD, and female E3FAD mice treated in PVT paradigm. Images taken at 20x magnification.

**Fig. S12** RVS paradigm: Representative close-up images of GFAP immunostaining (Green, scale bar: 500µm) in the (**A**) CX and the (**B**) HP female E4FAD, male E4FAD, and female E3FAD mice treated in RVS paradigm. Images taken at 20x magnification.

**Fig. S13** RVS paradigm: Representative close-up images of Iba-1 immunostaining (Green, scale bar: 500µm) in the (**A**) CX and the (**B**) HP female E4FAD, male E4FAD, and female E3FAD mice treated in RVS paradigm. Images taken at 20x magnification.

**Fig. S14** RVS paradigm: Representative close-up images of MOAB-2 immunostaining (Red, scale bar: 500µm) in the (**A**) CX and the (**B**) HP female E4FAD, male E4FAD, and female E3FAD mice treated in RVS paradigm. Images taken at 20x magnification.

**Fig. S15** RVS paradigm: Representative close-up images of Thio-S immunostaining (Green, scale bar: 500µm) in the (**A**) CX and the (B) HP female E4FAD, male E4FAD, and female E3FAD mice treated in RVS paradigm. Images taken at 20x magnification.
