# Supplementary material for: A small-molecule TLR4 antagonist reduced neuroinflammation in female E4FAD mice
Source: Alzheimers Res Ther. 2023 Oct 19;15:181. doi: 10.1186/s13195-023-01330-6 (PMC10585767; doi:10.1186/s13195-023-01330-6)
Supplement: Supplementary file 2 — Additional file 2: Table 1. List of reagents and antibodies. [file 13195_2023_1330_MOESM2_ESM.docx]

**Table 1. List of reagents and antibodies**
